# A randomized phase III study of short-course radiotherapy combined with Temozolomide in elderly patients with newly diagnosed glioblastoma; Japan clinical oncology group study JCOG1910 (AgedGlio-PIII)

**DOI:** 10.1186/s12885-021-08834-0

**Published:** 2021-10-15

**Authors:** Yoshiki Arakawa, Keita Sasaki, Yohei Mineharu, Megumi Uto, Takashi Mizowaki, Junki Mizusawa, Yuta Sekino, Tomohiro Ono, Hidefumi Aoyama, Kaishi Satomi, Koichi Ichimura, Manabu Kinoshita, Makoto Ohno, Yoshinori Ito, Ryo Nishikawa, Haruhiko Fukuda, Yasumasa Nishimura, Yoshitaka Narita, Brain Tumor Study Group and Radiation Therapy Study Group of the Japan Clinical Oncology Group

**Affiliations:** 1 Department of Neurosurgery, Kyoto University Graduate School of Medicine, 54 Kawaharacho, Shogoin, Sakyo-ku, Kyoto, 606-8507 Japan; 2 JCOG Data Center/Operations Office, National Cancer Center Hospital, Tokyo, Japan; 3 Department of Radiation Oncology and Image-Applied Therapy, Kyoto University Graduate School of Medicine, 54 Kawaharacho, Shogoin, Sakyo-ku, Kyoto, 606-8507 Japan; 4 Department of Radiation Oncology, Faculty of Medicine, Hokkaido University, Sapporo, Hokkaido Japan; 5 Department of Diagnostic Pathology, National Cancer Center Hospital, Tokyo, Japan; 6 Department of Brain Disease Translational Research, Juntendo University Faculty of Medicine, Tokyo, Japan; 7 Department of Neurosurgery, Asahikawa Medical University, Asahikawa, Hokkaido Japan; 8 Department of Neurosurgery and Neuro-Oncology, National Cancer Center Hospital, Tokyo, Japan; 9 Department of Radiation Oncology, Showa University Graduate School of Medicine, Tokyo, Japan; 10 Department of Neuro-Oncology/Neurosurgery, Saitama Medical University International Medical Center, Saitama, Japan; 11 Department of Radiation Oncology, Kindai University Faculty of Medicine, Osaka, Japan

**Keywords:** Randomized controlled trial, Glioblastoma, Elderly, Temozolomide, Short-course radiotherapy

## Abstract

**Background:**

The current standard treatment for elderly patients with newly diagnosed glioblastoma is surgery followed by short-course radiotherapy with temozolomide. In recent studies, 40 Gy in 15 fractions vs. 60 Gy in 30 fractions, 34 Gy in 10 fractions vs. 60 Gy in 30 fractions, and 40 Gy in 15 fractions vs. 25 Gy in 5 fractions have been reported as non-inferior. The addition of temozolomide increased the survival benefit of radiotherapy with 40 Gy in 15 fractions. However, the optimal regimen for radiotherapy plus concomitant temozolomide remains unresolved.

**Methods:**

This multi-institutional randomized phase III trial was commenced to confirm the non-inferiority of radiotherapy comprising 25 Gy in 5 fractions with concomitant (150 mg/m^2^/day, 5 days) and adjuvant temozolomide over 40 Gy in 15 fractions with concomitant (75 mg/m^2^/day, every day from first to last day of radiation) and adjuvant temozolomide in terms of overall survival (OS) in elderly patients with newly diagnosed glioblastoma. A total of 270 patients will be accrued from 51 Japanese institutions in 4 years and follow-up will last 2 years. Patients 71 years of age or older, or 71–75 years old with resection of less than 90% of the contrast-enhanced region, will be registered and randomly assigned to each group with 1:1 allocation. The primary endpoint is OS, and the secondary endpoints are progression-free survival, frequency of adverse events, proportion of Karnofsky performance status preservation, and proportion of health-related quality of life preservation. The Japan Clinical Oncology Group Protocol Review Committee approved this study protocol in April 2020. Ethics approval was granted by the National Cancer Center Hospital Certified Review Board. Patient enrollment began in August 2020.

**Discussion:**

If the primary endpoint is met, short-course radiotherapy comprising 25 Gy in 5 fractions with concomitant and adjuvant temozolomide will be a standard of care for elderly patients with newly diagnosed glioblastoma.

**Trial registration:**

Registry number: jRCTs031200099.

Date of Registration: 27/Aug/2020. Date of First Participant Enrollment: 4/Sep/2020.

## Background

Glioblastoma is an incurable primary brain tumor with a median overall survival (OS) less than 2 years and a 5-year survival rate of 7.2–16%, and comprising 12–14.5% of primary brain tumors [1, 2]. The incidence of glioblastoma peaks among patients in their 60s to 70s and is increasing with the growth of the elderly population [1–3]. Age is associated with poor prognosis, and median OS is less than 12 months among patients over 70 years old [1]. Initial Karnofsky performance status (KPS) before treatment is lower than 70 in 36% of patients [1]. Furthermore, the progression of glioblastoma can easily decrease performance status by reducing functional and cognitive ability, particularly among elderly patients.

The present standard treatment for patients younger than 70 years old with glioblastoma is radiotherapy comprising 60 Gy in 30 fractions with concomitant and adjuvant temozolomide [4, 5]. Most clinical trials for glioblastoma have excluded elderly patients because of their poor prognosis, comorbidity and sensitivity of the aging brain to radiation [6]. Several studies of elderly patients with glioblastoma evaluating radiotherapy as 60 Gy in 30 fractions with concomitant and adjuvant temozolomide showed prolonged OS in patients with good performance status but treatment-related toxicities such as greater deterioration of mental status [6–9]. Although conventionally fractionated radiotherapy effectively prolonged survival in elderly patients with glioblastoma, low completion rates due to the long duration of treatment and declines in activities of daily living (ADL) remain concerning. Hypofractionated radiotherapy has thus been developed to preserve efficacy and decrease toxicities during treatment.

In 2017, the Canadian Cancer Clinical Trials Group (CCTG) and the European Organization for Research and Treatment of Cancer (EORTC) reported that median OS was longer with short-course radiotherapy comprising 40 Gy in 15 fractions plus concomitant (75 mg/m^2^, daily from first to last day of radiation) and adjuvant (150–200 mg/m^2^, days 1–5, every 4 weeks, 12 cycles) temozolomide than with 40 Gy in 15 fractions alone (9.3 months vs. 7.6 months), as was median progression-free survival (PFS) (5.3 months vs. 3.9 months) in elderly patients with newly diagnosed glioblastoma (CE.6) [10]. The addition of temozolomide to short-course radiotherapy did not disturb quality of life (QOL) within manageable chemotherapy-related adverse events. However, in a subgroup analysis, patients 65–70 years old benefited less from the addition of temozolomide than those 71–75 years old or 76 years old or older. In a background with fewer enrolled patients 65 to 70 years old than 71 to 75 years old, patients 65 to 70 years old with satisfactory performance status are generally treated with radiotherapy as 60 Gy in 30 fractions plus concomitant and adjuvant temozolomide. Based on these considerations, a regimen of radiotherapy as 40 Gy in 15 fractions plus temozolomide could be an appropriate standard treatment for newly diagnosed glioblastoma patients 71 years old or above.

Three other phase III trials were conducted for further hypofractionated radiotherapy in elderly patients with newly diagnosed glioblastoma. Those studies compared 40 Gy in 15 fractions vs. 60 Gy in 30 fractions [11], 34 Gy in 10 fractions vs. 60 Gy in 30 fractions [12], and 40 Gy in 15 fractions vs. 25 Gy in 5 fractions [13] and reported non-inferior results in terms of safety and efficacy for elderly patients with newly diagnosed glioblastoma (Table 1 [10–15]). However, those studies all had small sample sizes and the results have not been confirmed by other investigations, so the optimal dose and number of fractions remain unclear, particularly when administered in combination with temozolomide.

**Table 1:** Randomized controlled trials in elderly patients with glioblastoma

Authors, publication year, study name	Age cut-off, years	Number	Intervention	mPFS, months	mOS, months	
Roa et al., 2004 [11]	≥60	47	60 Gy/30 fr	N/A	5.1	
		48	40 Gy/15 fr	N/A	5.6	
Keime-Guibert et al., 2007 [14], ANOCEF	≥70	42	Best supportive care	1.2	3.9	
		39	50 Gy/28 fr	3.4	6.7	
Wick et al., 2012 [15], NOA-08	> 65	178	60 Gy/30 fr	4.7	9.6	
		195	Dose-dense temozolomide	3.3	8.6	
Malmström et al., 2012 [12], Nordic study	> 70	100	60 Gy/30 fr	N/A	6	
		98	34 Gy/10 fr	N/A	7.5	
		93	Temozolomide	N/A	8.3	
Roa et al., 2015 [13]	≥65	50	40 Gy/15 fr	4.2	6.4	
		48	25 Gy/5 fr	4.2	7.9	
Perry et al., 2017 [10], CE.6	≥65	281	40 Gy/15 fr	3.9	7.6	
		281	40 Gy/15 fr + temozolomide	5.3	9.3	

*mPFS* median progression-free survival; *mOS* median overall survival; *fr* fractions; *N/A* not available

In August 2020, the Brain Tumor Study Group and the Radiation Therapy Study Group of the Japan Clinical Oncology Group (JCOG) started a multicenter randomized controlled phase III trial in elderly patients with newly diagnosed glioblastoma (JCOG1910, AgedGlio-PIII).

## Methods / design

### Aim

The purpose of this study was to confirm the non-inferiority of radiotherapy as 25 Gy in 5 fractions with concomitant and adjuvant temozolomide over 40 Gy in 15 fractions with concomitant and adjuvant temozolomide in terms of OS in elderly patients with newly diagnosed glioblastoma.

### Randomization

The first registration is performed by JCOG Web System to the JCOG Data Center if all eligibility criteria for the first registration are satisfied. Methylation status of the O6-methylguanine-DNA methyltransferase (MGMT) promoter region is examined using surgical specimens before the second registration. After confirming the inclusion criteria for the second registration have been met, the second registration will be made by the JCOG Web System to the JCOG Data Center. Patients will be randomized in a 1:1 ratio into either arm A (standard treatment arm: radiotherapy as 40 Gy in 15 fractions with concomitant and adjuvant temozolomide) or arm B (experimental treatment arm: radiotherapy as 25 Gy in 5 fractions with concomitant and adjuvant temozolomide) using a minimization method with a random component while balancing arms with respect to institution, extent of surgical resection (partial resection + total resection vs. biopsy), Karnofsky performance status (< 70% vs. ≥70%) and methylation status of the MGMT promoter region (methylated vs. unmethylated).

### Study setting

A multi-institutional, randomized controlled trial.

### Funding

This study is supported by the National Cancer Center Research and Development Fund (2020-J-3) and the Japan Agency for Medical Research and Development (JP20ck0106619).

### Endpoints

The primary endpoint is OS, and secondary endpoints are PFS, frequency of adverse events (AEs), proportion of Karnofsky performance status preservation, and proportion of health-related QOL preservation.

OS is defined as the time from registration to death from any cause, censored as of the last day when the patient is known to be alive. PFS is defined as the time from registration to either the first event of tumor progression or death from any cause, censored as of the latest day when the patient is alive without any evidence of progression. Tumor response is evaluated according to the Response Assessment in Neuro-Oncology (RANO) criteria [16]. AEs are evaluated according to the Common Terminology Criteria for Adverse Events version 5.0 (CTCAE 5.0). SAE is defined as any ≥ grade 4 non-hematological toxicity at least possibly related to treatment, unexpected AE requiring hospitalization or prolongation of hospital stay, death from any cause during treatment or within 30 days after a protocol treatment, or treatment-related death. Health-related QOL is measured in accordance with EORTC QLQ-C30 and EORTC QLQ-BN20.

### Inclusion criteria

First registration criteria:

1. Tumor diagnosed as glioblastoma or Grade III glioma (WHO 2016 criteria) in pathological diagnosis during surgery, or histologically proven glioblastoma diagnosed according to WHO 2016 criteria.
2. Tumor specimen available for analysis of MGMT promoter methylation status.
3. No history of treatments for glioma except for resection including biopsy; in that case, second resection within 21 days after first resection.
4. First registration within 21 days after surgery.
5. Tumor present in the supratentorial region on preoperative contrast-enhanced MRI of the brain.
6. Preoperative contrast-enhanced MRI of the brain reveals no dissemination.
7. Presence of a measurable lesion is not mandatory.
8. Patient 71 years old or older at registration; 71 to 75 years old with resection of less than 90% of contrast-enhanced region.
9. ECOG performance status (PS) of 0, 1, 2, or 3 due to neurological symptoms caused by the tumor.
10. No prior chemotherapy or radiation therapy to the brain for any intracranial tumors.
11. Sufficient organ function.

(i) Neutrophil count ≥1500 /mm^3^.
(ii) Hemoglobin ≥8.0 mg/dl.
(iii) Platelet count ≥100,000 /mm^3^.
(iv) AST ≤ 120 U/L.
(v) ALT ≤120 U/L.
(vi) Cr ≤ 1.605 mg/dL for males, or ≤ 1.185 mg/dL for females.

(12) Informed consent provided voluntarily by the patient.

Second registration criteria:

1. Second registration within 21 days of the first registration.
2. Histologically proven glioblastoma diagnosed according to WHO 2016 criteria.
3. Confirmation of methylation or unmethylation in MGMT promoter region.

Exclusion criteria

1. Synchronous or metachronous malignancies (within the preceding 2 years).
2. Infections needing systemic treatment at time of registration.
3. Body temperature above 38 °C at registration.
4. Severe psychological disease at time of registration.
5. Continuous systemic corticosteroid or immunosuppressant treatment due to diseases other than brain tumor.
6. Uncontrollable diabetes mellitus.
7. Unstable angina pectoris, or history of myocardial infarction within the preceding 6 months.
8. Interstitial pneumonia, pulmonary fibrosis, or severe emphysema at time of registration.
9. Gadolinium allergy.
10. Positive results for HIV antibody.
11. Positive results for HBs antigen.

### Interventions

#### Arm A:

(1) 40 Gy in 15 daily fractions with temozolomide.

(i) Concomitant phase, temozolomide (75 mg/m^2^, daily from first to last day of radiation), radiation (2.67 Gy/day, 5 days/week, 15 times and 40 Gy in total)

(ii) Maintenance phase, temozolomide (150–200 mg/m^2^, days 1–5, every 4 weeks) 12 cycles.

#### Arm B:

(1) 25 Gy in 5 daily fractions with temozolomide.

(i) Concomitant phase, temozolomide (150 mg/m^2^, 5 days from first day), radiation (5 Gy/day, 5 days/week, 5 times and 25 Gy in total)

(ii) Maintenance phase, temozolomide (150–200 mg/m^2^, days 1–5, every 4 weeks) 12 cycles.

### Procedures of radiotherapy

Contouring was performed using computed tomography (CT) with a maximum slice thickness of 5 mm. Gross tumor volume (GTV) was defined as residual tumor according to pre- and postoperative contrast-enhanced MRI. Clinical target volume (CTV) was created by adding 1.5-cm margins to the GTV and resection cavity. In addition, CTV included surrounding edema (high-intensity area on T2-weighted or fluid-attenuated inversion recovery images). The margin for the planning target volume was set at 3–5 mm. The same target setting was used for Arms A and B.

### Follow-up

All patients will be followed-up for at least 2 years after completion of protocol treatment. Gadolinium-enhanced MRI of the brain will be evaluated at least every 8 weeks until disease progression or death. Tumor response will also be assessed every 8 weeks according to RANO criteria. Physical examination, laboratory tests, and evaluation of AEs according to CTCAE version 5.0 will be carried out at least every 4 weeks during protocol treatment.

### Statistical analysis

This study is designed as a multi-institutional, randomized controlled trial to confirm the non-inferiority of radiotherapy of 25 Gy in 5 fractions with concomitant and adjuvant temozolomide over 40 Gy in 15 fractions with concomitant and adjuvant temozolomide in terms of OS in elderly patients with newly diagnosed glioblastoma. The required sample size for randomization is calculated as 264, which will provide 70% power with a hypothesized 1-year survival rate of 37.8% in both arms and a non-inferiority margin of 1.32 using a one-sided alpha of 0.05 to observe 249 deaths. Considering some patients will not be registered due to progression before the second registration, the planned sample size for the first registration is 270 patients in a 4-year accrual period and 2-year follow-up period. Stratified Cox regression analysis with extent of surgical resection, Karnofsky performance status and methylation status of the MGMT promoter region will be performed for primary analysis.

### Interim analysis and monitoring

We plan to conduct an interim analysis to judge whether the study should be terminated early due to futility or clear evidence of efficacy. The interim analysis will be conducted after half of the planned number patients have been enrolled. The Lan-DeMets method with an O’Brien and Fleming-type alpha spending function will be used to adjust the multiplicity of the interim analysis and the primary analysis [17].

JCOG Data Center staff will conduct central monitoring issuing a monitoring report twice annually to evaluate and improve study progress, data integrity and patient safety. Futility will be considered at each monitoring report if necessary. For quality assurance, site-visit audits, not for a study-specific basis but for the study group basis, will be performed by the JCOG Audit Committee.

## Discussion

Reducing the treatment burden while maintaining efficacy is important for elderly patients with newly diagnosed glioblastoma, which shows poor prognosis. The disadvantages of the standard treatment are prolonged gastrointestinal symptoms caused by temozolomide, prolonged hospitalization for about 2 months, and deterioration of QOL due to hospitalization. This study therefore plans to confirm whether hypofractionated radiotherapy requiring a shorter treatment period with temozolomide can overcome the disadvantages of standard treatment without compromising efficacy.

Based on the CE.6 trial results, the regimen of radiotherapy comprising 40 Gy in 15 fractions plus temozolomide is the standard treatment for newly diagnosed glioblastoma patients 65 years old or older. In actual clinical practice, however, elderly patients with satisfactory performance status, particularly those under 75 years old, tend to be treated with 60 Gy of radiotherapy in 30 fractions plus concomitant and adjuvant temozolomide. Our other phase III trial, JCOG1703, began in June 2019 and is currently recruiting patients under 75 years old with newly diagnosed glioblastoma to confirm the superiority of maximal resection with carmustine wafer implantation followed by chemoradiotherapy with temozolomide over maximal resection followed by chemoradiotherapy with temozolomide, at least in terms of OS [18]. As the definition of maximal tumor resection is resection exceeding 90%, patients with 71–75 years and resection of less than 90% of the contrast-enhanced region has been set as an inclusion criterion for this study.

*MGMT* is a DNA repair enzyme that confers chemoresistance to alkylating agents [19]. Promoter methylation of *MGMT* reflects epigenetic silencing associated with longer survival in patients with glioblastoma treated using alkylating agents. In the analysis of the CE.3 trial, patients with a methylated *MGMT* promoter showed a survival benefit from temozolomide (median OS, 21.7 months vs. 15.3 months), whereas those with an unmethylated *MGMT* promoter did not (median OS, 12.7 months vs. 11.8 months) [20]. Furthermore, *MGMT* promoter methylation represents an independent favorable prognostic factor irrespective of treatment. The CE.6 trial showed a similar result that patients with a methylated *MGMT* promoter achieved significantly prolonged survival with temozolomide (median OS, 13.5 months with radiotherapy plus temozolomide vs. 7.7 months with radiotherapy alone) and those with unmethylated *MGMT* promoter benefited slightly from temozolomide (median OS, 10.0 months with radiotherapy plus temozolomide vs. 7.9 months with radiotherapy alone) [10], which may suggest that short-course radiotherapy increases the efficacy of temozolomide in glioblastomas with unmethylated *MGMT* promoter.

Radiotherapeutic regimens of 40 Gy in 15 fractions, 34 Gy in 10 fractions, and 25 Gy in 5 fractions have been shown to offer similar efficacy and safety for elderly patients with glioblastoma according to the three earlier phase III studies [11–13]. The current task is to determine the optimal dose and number of fractions, particularly in combination with temozolomide, for elderly patients with newly diagnosed glioblastoma. The linear-quadratic (LQ) model has been used for predicting radiobiological response [21]. Equivalent-dose fractionation can be estimated by calculating equivalent doses in 2-Gy fractions (EQD2) on the LQ model. Although several studies have shown that the α/β values of glioma and normal brain tissue were 5–10 Gy and 2–3 Gy, respectively [22–24], and the α/β value of glioblastoma in elderly patients is estimated as less than 1.2 Gy from the results of recent clinical trials [10, 12, 13](Table 2). On the basis of the estimated α/β value 1.2 Gy, EQD2s of 40 Gy in 15 fractions and 25 Gy in 5 fractions were considered equivalent for glioblastoma in elderly patients, suggesting similar efficacy. Meanwhile, the EQD2 of 25 Gy in 5 fractions is less than that of 40 Gy in 15 fractions for normal brain tissue, meaning less damage would be incurred by 25 Gy in 5 fractions. These estimates are supported by study results finding no differences in OS, PFS, or QOL between patients receiving the two radiotherapy regimens of 40 Gy in 15 fractions and 25 Gy in 5 fractions [13].

**Table 2:** Equivalent dose in 2-Gy fractions (EQD2) estimated on the LQ model

		α/β value			
Clinical trials	Radiation dose/fractions	1.2	3.0	5.6	10.0
Conventional radiotherapy	60 Gy/30 fr	60.0	60.0	60.0	60.0
Perry et al., 2017 [10], CE.6	40 Gy/15 fr	48.4	45.4	43.6	42.3
Roa et al., 2015 [13]	25 Gy/5 fr	48.4	40.0	34.9	31.3
Malmström et al., 2012 [12], Nordic study	34 Gy/10 fr	48.9	43.5	40.3	38.0

As concomitant treatment in the standard treatment, temozolomide is administered at 75 mg/m^2^ for 21 days, resulting in a total dose of 1575 mg/m^2^. On the other hand, if temozolomide at 75 mg/m^2^ is adopted as the concomitant treatment, the total dose of temozolomide is considerably reduced to 375 mg/m^2^ in 5 days and treatment intensity may thus be insufficient. Administration of temozolomide at 150 mg/m^2^ for 5 days has been confirmed as safe and effective as the first administration of maintenance treatment in the previous report [25]. In the study treatment, a synergistic effect is expected because both the single dose of temozolomide and radiation are increased, and maintenance treatment is implemented 2 weeks earlier than that in the standard treatment, so that the efficacy of temozolomide treatment is considered to be enhanced. According to the above, temozolomide at 150 mg/m^2^/day administered for 5 days was adopted as concomitant treatment for the study treatment.

Based on the prediction of radiobiological response from the results of clinical trials, the aim of this study is to confirm the non-inferiority of radiotherapy comprising 25 Gy in 5 fractions with concomitant and adjuvant temozolomide over 40 Gy in 15 fractions with concomitant and adjuvant temozolomide in terms of OS among elderly patients with newly diagnosed glioblastoma. If the primary endpoint is met, radiotherapy as 25 Gy in 5 fractions with concomitant and adjuvant temozolomide will be established as a standard of care for elderly patients with newly diagnosed glioblastoma. Furthermore, the α/β value of glioblastoma in elderly patients will be confirmed as less than 1.2 Gy. This study is designed as a multi-institutional, randomized controlled trial with a non-inferiority margin of 1.32, considering the disease rarity.

## Data Availability

Data sharing is not applicable for this article, as no datasets have been generated or analyzed at the time of submission.

## Abbreviations

ADL: Activities of daily living
AEs: Adverse events
CCTG: Canadian Cancer Clinical Trials Group
CI: Confidence interval
CTCAE: Common Terminology Criteria for Adverse Events
ECOG: Eastern Cooperative Oncology Group
EORTC: European Organisation for Research and Treatment of Cancer
EQD2: Equivalent dose in 2-Gy fractions
JCOG: Japan Clinical Oncology Group
KPS: Karnofsky performance status
LQ: Linear-quadratic
MGMT: O6-methylguanine–DNA methyltransferase
MR: Magnetic resonance
OS: Overall survival
PFS: Progression-free survival
PS: Performance status
QOL: Quality of life
RANO: Response Assessment in Neuro-Oncology
SAE: Serious adverse event
SPIRIT: Standard Protocol Items: Recommendations for Interventional Trials
